# Comparative Effectiveness of Baloxavir Marboxil and Oseltamivir Treatment in Reducing Household Transmission of Influenza: A Post Hoc Analysis of the BLOCKSTONE Trial

**DOI:** 10.1111/irv.13302

**Published:** 2024-05-06

**Authors:** Hideyuki Ikematsu, Takamichi Baba, Masaya M. Saito, Masahiro Kinoshita, Shogo Miyazawa, Ayano Hata, Saki Nakano, Yoshitake Kitanishi, Frederick G. Hayden

**Affiliations:** ^1^ Ricerca Clinica Fukuoka Japan; ^2^ Biostatistics Center Shionogi & Co., Ltd Osaka Japan; ^3^ Department of Information Security University of Nagasaki Nagasaki Japan; ^4^ Medical Affairs Department Shionogi & Co., Ltd Osaka Japan; ^5^ Data Science Department Shionogi & Co, Ltd Osaka Japan; ^6^ Division of Infectious Diseases and International Health University of Virginia School of Medicine Virginia USA

**Keywords:** baloxavir marboxil, household transmission, influenza treatment, Japan, oseltamivir

## Abstract

**Background:**

The transmission of influenza virus in households, especially by children, is a major route of infection. Prior studies suggest that timely antiviral treatment of ill cases may reduce infection in household contacts. The aim of the study was to compare the effects of oseltamivir (OTV) and baloxavir marboxil (BXM) treatment of index cases on the secondary attack rate (SAR) of influenza within household.

**Methods:**

A post hoc analysis was done in BLOCKSTONE trial—a placebo‐controlled, double‐blinded post‐exposure prophylaxis of BXM. Data were derived from the laboratory‐confirmed index cases' household contacts who received placebo in the trial and also from household members who did not participate in the trial but completed illness questionnaires. To assess the SAR of household members, multivariate analyses adjusted for factors including age, vaccination status, and household size were performed and compared between contacts of index cases treated with BXM or OTV.

**Results:**

In total, 185 index cases (116 treated with BXM and 69 treated with OTV) and 410 household contacts (201 from trial, 209 by questionnaire) were included. The Poisson regression modeling showed that the SAR in household contacts of index cases treated with BXM and OTV was 10.8% and 18.5%, respectively; the adjusted relative reduction in SAR was 41.8% (95% confidence interval: 1.0%–65.7%, *p* = 0.0456) greater with BXM than OTV. Similar reductions were found in contacts from the trial and those included by questionnaire.

**Conclusion:**

BXM treatment of index cases appeared to result in a greater reduction in secondary household transmission than OTV treatment.

## 1 Introduction

Seasonal influenza has a global impact on populations and healthcare systems with as many as 1 billion infections, 1–3 million severe cases, and 650,000 deaths worldwide each year [1, 2]. During the Japanese 2018/2019 season, it was estimated that up to 12 million people visited medical facilities for influenza, leading to approximately 20,000 severe cases and more than 3400 excess deaths [3]. Household transmission of influenza virus is a major route of infection [4]. The spread of the infection from preschool‐ (≤ 4 years old) and school‐aged children (5–18 years old) to other family members is particularly high [4, 5, 6, 7]. Because of the magnitude of epidemics and their associated social disruption, effective control of influenza requires both non‐pharmaceutical public health measures and pharmaceutical interventions.

The secondary attack rate (SAR), defined as the rate of influenza transmission from the index case to household contacts, is useful for evaluating the effectiveness of various antiviral drugs in reducing the rate of intra‐household influenza transmission. Except for one oseltamivir (OTV) trial showing an approximate 20% reduction in influenza‐like illness SAR [8], observational studies of antiviral treatment of household index cases have demonstrated variable evidence for transmission reduction [7, 9, 10, 11].

Single‐dose baloxavir marboxil (BXM), a selective influenza cap‐dependent endonuclease inhibitor, shows more rapid and potent antiviral activity than OTV in treating both otherwise healthy and high‐risk adults, as well as pediatric influenza patients [12, 13, 14, 15]. One single‐center, retrospective observational study [16] and observational studies using a Japanese health insurance claims database maintained by the Japanese Medical Data Center [17, 18] have reported that BXM treatment is associated with relative risk reductions in SAR ranging from 25% to 51% compared to neuraminidase inhibitors (NAI). Also, a robust Phase 3 randomized controlled trial (RCT)—CENTERSTONE (NCT03969212)—is currently being conducted, but real‐word data or observational studies have also been utilized to gain insights on the effect of BXM on transmission.

The aim of the study was to compare the effects of OTV and BXM treatment of index patients on the SAR of influenza illness in household members. We have analyzed data from the BLOCKSTONE trial that demonstrated the high efficacy of post‐exposure prophylaxis against influenza illness in household contacts given single‐dose BXM compared to placebo [19]. Because antiviral treatment was given to all ill index cases, we have conducted a post hoc analysis of the prospectively collected household transmission data to assess the SARs in household contacts receiving placebo (or no antiviral) and who were exposed to index cases taking BXM or OTV treatment. Furthermore, we applied a previously reported model to estimate a detailed transmission profile for patients who had taken BXM or OTV [20].

## 2 Methods

### 2.1 Study Population and Monitoring

This study is based on data obtained in the BLOCKSTONE trial, a Phase 3, multicenter, double‐blind, randomized, placebo‐controlled trial that evaluated the post‐exposure prophylactic efficacy of BXM in household contacts of index cases with confirmed influenza during the 2018–2019 season in Japan [19]. All index cases within the trial had laboratory confirmation of influenza and had body temperature of 37.5°C or higher at screening. To evaluate the SAR in households, only households in which no one received BXM as prophylaxis were analyzed. The index cases received BXM or an NAI at the discretion of each investigator, so treatment for each index case was not randomized.

This study included data from both the household contacts in the BLOCKSTONE trial who were administered placebo and household contacts who did not participate in the BLOCKSTONE trial but responded to a questionnaire survey. The dates of illness onset and recovery of household contacts were determined using a self‐assessment diary. A paper‐based questionnaire survey to Day 15 was administered to household contacts who participated in the Phase 3 trial, and they or their guardians distributed the questionnaire to other household contacts. After informed consent was obtained from the household contacts or their legal guardian, demographic data and their illness status including the onset and recovery dates were recorded via the questionnaire survey (Data S1). The dataset of index cases infected with influenza A virus and household contacts including onset and recovery dates, as well as data on influenza‐like symptoms, was applied to a transmission profile simulation.

### 2.2 Endpoints

The definition of a secondary ill case included at least one of the following influenza‐like symptoms by Day 10 of the observation period: body temperature ≥ 37.5C°, cough, sore throat, headache, nasal discharge/nasal congestion, feverishness or chills, muscle or joint pain, and fatigue. For household contacts who participated in the trial, positive reverse transcriptase‐polymerase chain reaction (RT‐PCR) results with the same influenza subtype as in the index case were added to the definition of secondary infection. Virologic data were not available from those only completing the survey questionnaire. Recovery was defined as a body temperature < 37.0C°.

The primary endpoint of the current analysis was the SAR, which was defined as the number of newly ill cases divided by the number of susceptible household contacts. The secondary endpoint was the avoidance ratio of intra‐household ill cases, which was given as number of households in which no secondary ill cases occurred among household contacts divided by number of overall households. Other endpoints included serial interval, symptomatic period, incubation period, assumed infection period, and extended infection period, which were estimated using the transmission profile estimation model.

### 2.3 Statistical Analysis

Multivariate analysis was performed to adjust for the heterogeneity of the covariates in a comparison as a post hoc analysis, and the risk ratio was adopted as a parameter of group difference, as in the BLOCKSTONE trial [19]. The SAR was compared for index cases treated with BXM or OTV using the Poisson regression model, with the number of newly symptomatic persons in the household as the outcome; treatment of the index case as the fixed effect; age, influenza vaccination status within the previous 6 months of the index case and household size as covariates; and household size, except for the index case, as offsets. These covariates have been reported to be associated with secondary cases within a household [4, 5, 6, 7].

The avoidance ratios of intra‐household illness for BXM and OTV treatment were compared using a modified Poisson regression model [21], with the presence or absence of household transmission as the outcome and the same fixed effects and covariates as in the Poisson regression for SAR. Subgroup analysis for the comparison of the SAR and the avoidance ratios of intra‐household illness were conducted. In the subgroup analysis by influenza subtype (A/H1 and A/H3), age was excluded from the analysis model to avoid unstable estimation results due to no index case over 12 years old in OTV group for the subgroup of A/H1 illness. In addition, subgroup analysis of SAR by household contacts participating in the clinical trial or questionnaire survey were performed.

We also estimated the parameters describing the natural history of an infection using datasets of index cases and household contacts in the simulation‐applicable population. Symptomatic, infective, extended infective after recovery, incubation periods, and serial interval were estimated using the maximum likelihood procedure as previously reported [20]. A detailed description of these methods is provided in the Supporting Information.

All analyses were performed using SAS software, Version 9.4 (SAS Institute) and R Version 4.2.2.

## 3 Results

### 3.1 Characteristics of Index Cases and Contacts

**FIGURE 1:**
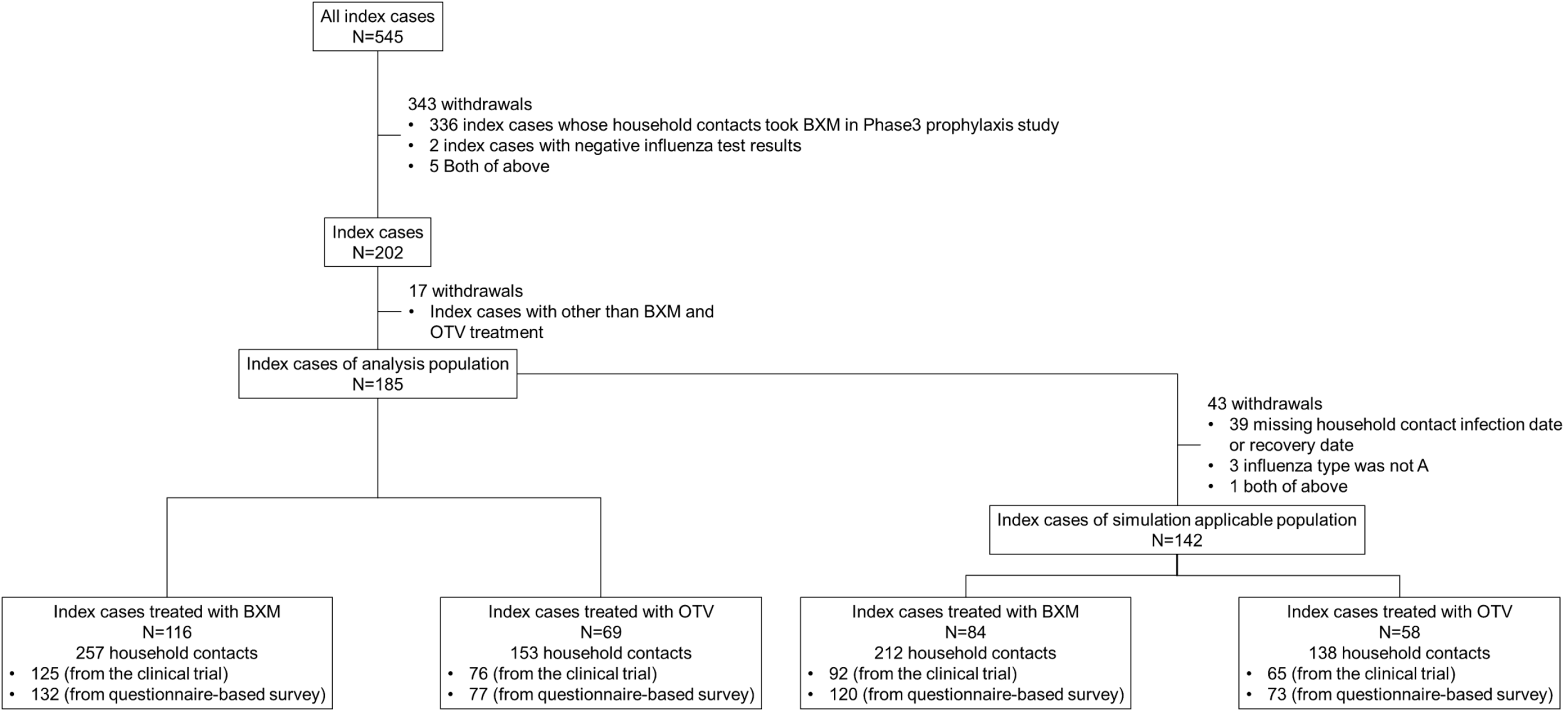
Index case disposition. BXM, baloxavir marboxil; OTV, oseltamivir.

The demographic characteristics of the index cases in the analysis population are shown in Table 1 and their disposition for the current analysis illustrated in Figure 1. A total of 185 index cases were identified in the BLOCKSTONE trial, 116 of whom were treated with BXM and 69 with OTV. Almost all (97.8%) had influenza A viral infection, 45.9% with A/H1 and 51.9% with A/H3. There was an imbalance in age between index case groups: BXM was prescribed to both patients aged < 12 years (53.4%) and above, whereas OTV prescription was prescribed primarily to patients aged < 12 years (94.2%). The other demographic characteristics were similar in the BXM and OTV groups. The demographic data of the index cases in simulation‐applicable population and household contacts of the index cases are listed in Tables S1 and S2, respectively.

**TABLE 1:** Demographic characteristics of the index cases.

		BXM (*N* = 116) *n* (%)	OTV (*N* = 69) *n* (%)	Overall (*N* = 185) *n* (%)
Sex	Male	63 (54.3)	37 (53.6)	100 (54.1)
Age	< 12 years	62 (53.4)	65 (94.2)	127 (68.6)
	≥ 12 to ≤ 64 years	49 (42.2)	3 (4.3)	52 (28.1)
	≥ 65 years	5 (4.3)	1 (1.4)	6 (3.2)
Vaccination^a^	Yes	38 (32.8)	25 (36.2)	63 (34.1)
Influenza virus type	Type A	112 (96.6)	69 (100.0)	181 (97.8)
	A/H1	53 (45.7)	32 (46.4)	85 (45.9)
	A/H3	59 (50.9)	37 (53.6)	96 (51.9)
	Type B	3 (2.6)	0	3 (1.6)
	Mixed infection	1 (0.9)	0	1 (0.5)
Occupation	Worker	14 (12.1)	2 (2.9)	16 (8.6)
	Student	97 (83.6)	60 (87.0)	157 (84.9)
	Neither	5 (4.3)	7 (10.1)	12 (6.5)
Household size except for index case excluded	< 3	34 (29.3)	19 (27.5)	53 (28.6)
	≥ 3	82 (70.7)	50 (72.5)	132 (71.4)

Abbreviations: BXM, baloxavir marboxil; OTV, oseltamivir.

^a^ Influenza vaccination status within the previous 6 months.

The total number of household contacts exposed to the 185 index cases in the analysis population was 536, of whom 410 (76.5%) participated in this study. Of the 410 household contacts (201 from trial, 209 by questionnaire), 257 and 153 were household contacts of BXM‐ and OTV‐treated index cases, respectively. The summary of demographics of the household contacts participating in the BLOCKSTONE trial, questionnaire survey, or overall was summarized in Table 2. Particularly, the distribution of time from symptom onset of index case to informed consent obtained from trial participants and study drug dosing in participants joining in BLOCKSTONE trial was similar in the BXM (72.8%) and OTV groups (77.6%). The demographic features of the household contacts showed no important differences.

**TABLE 2:** Demographic characteristics of the household contacts.

		Clinical trial		Questionnaire		Overall	
		BXM *N* = 125 *n* (%)	OTV *N* = 76 *n* (%)	BXM *N* = 132 *n* (%)	OTV *N* = 77 *n* (%)	BXM *N* = 257 *n* (%)	OTV *N* = 153 *n* (%)
Sex	Male	19 (15.2)	12 (15.8)	—	—	19 (7.4)	12 (7.8)
	Female	106 (84.8)	64 (84.2)	—	—	106 (41.2)	64 (41.8)
	Missing	—	—	132 (100.0)	77 (100.0)	132 (51.4)	77 (50.3)
Age	< 12 years	6 (4.8)	6 (7.9)	11 (8.3)	19 (24.7)	17 (6.6)	25 (16.3)
	≥ 12 to ≤ 64 years	110 (88.0)	66 (86.8)	11 (8.3)	3 (3.9)	121 (47.1)	69 (45.1)
	≥ 65 years	9 (7.2)	4 (5.3)	0	0	9 (3.5)	4 (2.6)
	Missing	—	—	110 (83.3)	55 (71.4)	110 (42.8)	55 (35.9)
Time from onset of influenza virus infection of index case to informed consent of household contact	< 24 h	91 (72.8)	59 (77.6)	—	—	91 (35.4)	59 (38.6)
	≥ 24 to ≤ 48 h	34 (27.2)	17 (22.4)	—	—	34 (13.2)	17 (11.1)
	Missing	—	—	132 (100.0)	77 (100.0)	132 (51.4)	77 (50.3)
Vaccination^a^	Yes	44 (35.2)	27 (35.5)	—	—	44 (17.1)	27 (17.6)
	No	81 (64.8)	49 (64.5)	—	—	81 (31.5)	49 (32.0)
	Missing	—	—	132 (100.0)	77 (100.0)	132 (51.4)	77 (50.3)
Influenza virus type of index case	Type A	121 (96.8)	76 (100.0)	131 (99.2)	77 (100.0)	252 (98.1)	153 (100.0)
	A/H1	58 (46.4)	36 (47.4)	47 (35.6)	42 (54.5)	105 (40.9)	78 (51.0)
	A/H3	63 (50.4)	40 (52.6)	84 (63.6)	35 (45.5)	147 (57.2)	75 (49.0)
	Type B	3 (2.4)	0	0	0	3 (1.2)	0
	Mixed infection	1 (0.8)	0	1 (0.8)	0	2 (0.8)	0
Occupation	Worker	81 (64.8)	52 (68.4)	7 (5.3)	2 (2.6)	88 (34.2)	54 (35.3)
	Student	7 (5.6)	7 (9.2)	13 (9.8)	16 (20.8)	20 (7.8)	23 (15.0)
	Neither	37 (29.6)	17 (22.4)	2 (1.5)	5 (6.5)	39 (15.2)	22 (14.4)
	Missing	—	—	110 (83.3)	54 (70.1)	110 (42.8)	54 (35.3)

*Note:* Age and occupation of household contacts who participated in the questionnaire survey were available if they were ill.

Abbreviations: BXM, baloxavir marboxil; OTV, oseltamivir.

^a^ Influenza vaccination status within the previous 6 months.

### 3.2 Secondary Attack Rates

**FIGURE 2:**
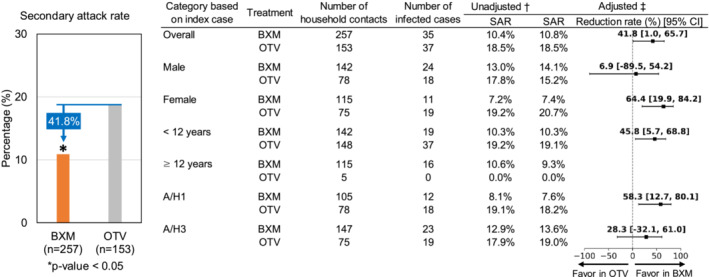
Secondary attack rate based on Poisson regression model. BXM, baloxavir marboxil; CI, confidence interval; OTV, oseltamivir; SAR, secondary attack rate. BXM was used as a reference against OTV to calculate the SAR rate. ^†^Poisson regression model with the number of newly ill persons in the household as the outcome, treatment of the index case as the fixed effect, and household size, except for the index case, as offsets. ^‡^Poisson regression model with the number of newly ill persons in the household as the outcome, treatment of the index case as the fixed effect, age, influenza vaccination status within the previous 6 months of the index case and household size as covariates, and household size, except for the index case excluded, as offsets. In subgroup analysis by age and influenza subtype, age was excluded from the analysis model.

The results of the Poisson regression modeling of the SAR are shown in Figure 2. The adjusted overall SARs were 10.8% for household contacts of the index cases treated with BXM and 18.5% for those treated with OTV. The relative reduction of adjusted SAR was 41.8% (95% confidence interval [CI]: 1.0%–65.7%, *p* = 0.0456). In the subgroup of index cases aged < 12 years, 142 were household contacts of 62 BXM‐treated index cases, and 148 were household contacts of 65 OTV‐treated index cases. The adjusted SARs were 10.3% and 19.1%, respectively, and the relative reduction of the contacts of index cases treated with BXM was 45.8% (95% CI: 5.7%–68.8%).

In addition, the relative reduction of adjusted SAR in female index cases (64.4%, 95% CI: 19.9%–84.2%) was much greater than that in male index cases (6.9%, 95% CI: −89.5%–54.2%). Furthermore, the relative reduction of adjusted SAR in index cases with A/H1 infection (58.3%, 95% CI: 12.7%–80.1%) was greater than that with A/H3 infection (28.3%, 95% CI: −32.1%–61.0%). Of note, the relative reduction of adjusted SAR in household contacts participating in the BLOCKSTONE trial and those participating in questionnaire survey was 35.4% (95% CI: −35.5–69.2%) and 42.0% (95% CI: −24.4%–73.0%), respectively (Table 3). The demographic characteristics of the ill household contacts participating in the BLOCKSTONE trial or questionnaire survey are shown in Table S3. Notably, the proportion of household contacts aged < 12 years in the group of questionnaire survey group (69.4%) was higher than that of BLOCKSTONE trial (2.8%).

**TABLE 3:** Secondary attack rate based on Poisson regression model of household contacts participating in clinical trial or questionnaire survey.

Population	Treatment	Number of household contacts	Number of ill cases	Unadjusted^a^	Adjusted^b^	
				SAR	SAR	Reduction rate (%) [95% CI]
Clinical trial	BXM	125	18	14.4%	15.3%	35.4 [−35.5, 69.2]
	OTV	76	18	23.7%	23.7%	—
Questionnaire	BXM	132	17	12.9%	12.0%	42.0 [−24.4, 73.0]
	OTV	77	19	24.7%	20.7%	—

*Note:* BXM was used as a reference against OTV to calculate the SAR rate. Clinical trial: Household contacts participated in BLOCKSTONE trial were enrolled. Questionnaire: Household contacts participated in this study via questionnaire survey were enrolled.

Abbreviations: BXM, baloxavir marboxil; CI, confidence interval; OTV, oseltamivir; SAR, secondary attack rate.

^a^ Poisson regression model with the number of newly ill persons among the population in the household as the outcome, treatment of the index case as the fixed effect, and household size among the population, except for the index case excluded, as offsets.

^b^ Poisson regression model with the number of newly ill persons among the population in the household as the outcome, treatment of the index case as the fixed effect, age, influenza vaccination status within the previous 6 months of the index case and household size as covariates, and household size among the population, except for the index case, as offsets.

### 3.3 Avoidance Ratio of Intra‐household Ill Cases

As shown in Figure S1, the adjusted avoidance ratios were 70.9% for household contacts of index cases treated with BXM and 57.7% for those of index cases treated with OTV. The relative increase of the avoidance ratio of households with BXM‐treated index cases was 23.0% (95% CI: −1.1%–53.0%, *p* = 0.0629). In the subgroup of index cases aged < 12 years, the adjusted avoidance ratios were 66.8% for the BXM group and 52.0% for the OTV group, with a relative increase of 28.5% (95% CI: 1.0%–63.4%). In other subgroups, adjusted avoidance ratios of BXM‐treated index cases was consistently higher than OTV‐treated index cases other than index cases aged ≥ 12 years due to small sample size.

### 3.4 Transmission Profile Simulation

The results of the estimation of the parameters related to the profile of transmission from the BXM‐ and OTV‐treated index cases are shown in Figure S2 and Table S4. The symptomatic period was similar for both treatment groups: 2.32 days (95% CI: 1.00–5.00 days) for BXM and 2.38 days (95% CI: 1.00–5.05 days) for OTV. Differences were seen in the serial interval: relatively larger number of secondary cases among household contacts in OTV at < 2 days was observed compared to that in BXM, but there was no difference in the median time to illness onset (3.00 days for both BXM and OTV). This serial interval profile for OTV translates to a shorter incubation period with a longer infective period, by a day each. The incubation and infective periods were, respectively, 1.62 (95% CI: 0.08–5.36) and 2.97 days for BXM and 0.72 (95% CI: 0–3.42) and 4.03 days for OTV. In addition, the symptomatic period was similar in both groups, and the infective period was longer for OTV. As a result, a longer extended infective period after recovery for OTV was observed: The extended infective periods after recovery were 0.65 and 1.65 days for BXM and OTV, respectively.

## 4 Discussion

This post hoc analysis of the BLOCKSTONE post‐exposure prophylaxis trial found that the SAR for household contacts of BXM‐treated index cases was significantly lower than that for household contacts of OTV‐treated index cases. The adjusted SARs for household contacts of the index cases treated with BXM were consistently lower than OTV in both household contacts enrolled in the BLOCKSTONE trial and the subgroup with only questionnaire data. The collection of household transmission information by questionnaire was used to collect information primarily from children who were unable to participate in the BLOCKSTONE trial. By collecting questionnaire‐based information on household transmission, it was realized to both verify the efficacy of BXM in post‐exposure prophylaxis and build evidence of BXM for reducing household transmission simultaneously in BLOCKSTONE trial. Our results are also directionally consistent with those of a previous study using a Japanese claims database for the 2018–2019 influenza season, which indicated that household transmission was 17.98% in the BXM group and 24.16% in the OTV group [17]. Our findings support the hypothesis that the more rapid decline in the infectious viral load with BXM than with OTV treatment [9, 10, 22, 23, 24] is associated with greater reduction in the risk of household influenza virus transmission.

It is well established that the frequency of secondary infections from younger children to other household members is high because children are more susceptible than older age groups [4, 5, 6, 7], often have more prolonged virus replication [25], and are more likely to expose others in the household related to behavior patterns and lower adherence to infection control practices [26]. We found that a nominally significant reduction consistently with the overall population in the adjusted SAR was observed in contacts of index cases aged < 12 years treated with BXM compared to OTV. One reason for this result may have been the difference in administration, which was a single dose of BXM versus twice daily for 5 days for OTV. Thus, BXM is expected to have higher compliance than OTV in children. Also, the duration of A/H1 virus isolation was reported to be longer in OTV‐treated patients than in BXM‐treated patients [27]. This may be one of the reasons behind the higher relative reduction of adjusted SAR in contacts of A/H1‐infected index cases. In addition, it was found that the relative reduction of adjusted SAR in female index cases was greater than that of male index cases. To explore the reasons, additional analysis was done as Table S5 to see SARs by sex and age. A possible cause was that the SAR for female < 12 years treated with BXM (5.8%) was lower than that of the other subgroups, but the number of index cases per subgroup was small. The reason for the gender difference of SAR remains unexplained and will need to be evaluated in larger studies.

The use of BXM as a treatment for children with influenza appears to reduce the risk of infection transmission in households, but the high frequency of treatment‐related emergence of BXM‐resistant variants, especially in children aged 5 years or less with influenza A/H3 illness [28], raises concerns. In this regard, although transmission of influenza A/H3 virus with PA/I38T substitutions conferring BXM resistance from BXM‐treated children to close contacts has been documented [29], the BLOCKSTONE trial did not detect BXM‐resistant variants in the household contacts receiving placebo who were exposed to BXM‐treated index cases [19, 30]. This finding suggests that the risk of transmission of such variants is relatively low in the household setting but requires continued study. In this regard, the effect of BXM treatment of index cases on reducing virus transmission to household contacts is currently being investigated in a large Phase 3 placebo‐controlled, double‐blind, randomized trial called CENTERSTONE (NCT03969212), which will evaluate the effect of BXM treatment on reducing virus transmission to household contacts and assess the susceptibility of breakthrough infections.

An estimation of the detailed infectious period based on a modified Cauchemez‐type model have been reported [20]. By applying this model, we observed that the incubation period with the OTV treatment was shorter than that with the BXM treatment. However, the incubation period was generally not affected by anti‐influenza drug treatment, and the small number of secondary cases may have led to large uncertainties in our estimations.

Our study had several limitations. First, the drug used to treat the index cases was not randomly assigned. Because there were few index cases with over 12 years of age given OTV treatment, multivariate analysis may not work well in adjusting for heterogeneity. Moreover, there may be unknown confounding factors that cannot be adjusted for. Second, the susceptibility of household contacts is related to their demographic characteristics [4, 5, 6, 7], but we could not obtain these data from those who only participated in the questionnaire survey. Third, the frequency of asymptomatic infections was not determined. Thus, the frequency of household transmission may be underestimated. Fourth, RT‐PCR testing was not performed during enrollment of household contacts, so some of them were likely already infected at enrollment, which would have led to an underestimation of group differences. Fifth, because not all household contacts of the index cases participated in this study, not all secondary patients were captured. Thus, the avoidance ratio of intra‐household ill cases may have been underestimated. Sixth, the transmission risk profile of index cases did not include data on duration of infectious virus detectability or the possible effect of influenza vaccination within the preceding 6 months. Similarly, the analysis of the possible impact of influenza vaccination on illness risk and severity in contacts was limited by small numbers. The ongoing CENTERSTONE trial should provide definitive evidence of the inhibitory effect of BXM treatment of index cases on household transmission and more data on its social and socioeconomic impacts.

In summary, this post hoc analysis found that the secondary influenza illness attack rate was lower in household contacts exposed to BXM‐treated than OTV‐treated index cases. In addition, this reduction was maintained in the subgroup of index cases aged < 12 years. Other modeling studies indicate that early treatment BXM could lead to substantial reductions in community transmission of influenza viruses and reduce its well‐recognized impacts [31, 32]. The more rapid reduction in infectious virus titers associated with BXM compared to OTV treatment may explain BXM's greater reduction in secondary household transmission than observed with OTV.

## Author Contributions

**Hideyuki Ikematsu:** conceptualization, investigation, writing–original draft, writing–review and editing. **Takamichi Baba:** conceptualization, formal analysis, writing–original draft, writing–review and editing. **Masaya M. Saito:** conceptualization, formal analysis, writing–original draft, writing–review and editing. **Masahiro Kinoshita:** conceptualization, writing–original draft, writing–review and editing. **Shogo Miyazawa:** formal analysis, writing–original draft, writing–review and editing. **Ayano Hata:** formal analysis, writing–original draft, writing–review and editing. **Saki Nakano:** formal analysis, writing–original draft, writing–review and editing. **Yoshitake Kitanishi:** conceptualization, writing–original draft, writing–review and editing. **Frederick G. Hayden:** conceptualization, investigation, writing–original draft, writing–review and editing.

## Ethics Statement

The trial was conducted in accordance with the principles of the Declaration of Helsinki and the Good Clinical Practice guidelines of the International Council for Harmonization. The protocol was approved by an institutional review board at each trial site.

## Consent

All the index cases and participating household contacts (or their legal representatives) provided written informed consent.

## Conflicts of Interest

H.I. and M.S. report no conflicts of interest. T.B., M.K., S.M., A.H., S.N., and Y.K. are employees of and hold stocks in Shionogi & Co., Ltd. F.G.H. reports serving as a non‐compensated consultant and recipient of meeting travel support from F. Hoffmann‐La Roche and Shionogi and serving as a non‐compensated consultant for Arcturus, Asterivir, Cidara Therapeutics, Fujifilm Corporation, Genentech, Gilead Sciences, Janssen Pharmaceuticals, Merck, MediVector, Ridgeback, SAB Biotherapeutics, Sanofi‐Pasteur, Versatope, Via Nova Therapeutics, Eradivir and Vir Biotechnology.

### Peer Review

The peer review history for this article is available at https://www.webofscience.com/api/gateway/wos/peer‐review/10.1111/irv.13302.

## Supporting information


**Figure S1.** Avoidance ratio of intra‐household ill cases based on modified Poisson regression model.
**Figure S2.** Serial interval and assumed infectious period in simulation‐applicable population.
**Table S1.** Demographic characteristics of the index cases (simulation‐applicable population).
**Table S2.** Demographic characteristics of the household contacts (simulation‐applicable population).
**Table S3.** Demographic characteristics of the ill household contacts who participating in the clinical trial or questionnaire survey.
**Table S4.** Estimated parameters in days for the transmission profiles of index cases in the simulation‐applicable population.
**Table S5.** Secondary attack rate based on Poisson regression model for household contacts participating in the clinical trial or questionnaire survey.

**Data S1** Supporting Information.

## Data Availability

Qualified researchers may request access to individual patient level data through the clinical study data request platform (https://vivli.org/). Further details on Roche's criteria for eligible studies are available here (https://vivli.org/members/ourmembers/). For further details on Roche's Global Policy on the Sharing of Clinical Information and how to request access to related clinical study documents, see here (https://www.roche.com/research_and_development/who_we_are_how_we_work/clinical_trials/our_commitment_to_data_sharing.htm).

## References

[1] World Health Organization , “WHO Launches New Global Influenza Strategy,” accessed February 21, 2023, https://www.who.int/news/item/11‐03‐2019‐who‐launches‐newglobal‐influenza‐strategy.

[2] Western Pacific Region Global Influenza Surveillance and Response System , “Epidemiological and Virological Characteristics of Influenza in the Western Pacific Region of the World Health Organization, 2006–2010,” PLoS ONE 7 (2012): e37568. 22675427 10.1371/journal.pone.0037568 PMC3366627

[3] NIID , “Influenza 2018/19 Season, Japan,” *IASR* Vol. 40 No.11, accessed February 21, 2023, https://www.niid.go.jp/niid/en/a‐h7n9‐en/865‐iasr/9288‐477te.html.

[4] B. J. Cowling , K. H. Chan , V. J. Fang , et al., “Comparative Epidemiology of Pandemic and Seasonal Influenza a in Households,” The New England Journal of Medicine 362 (2010): 2175–2184. 20558368 10.1056/NEJMoa0911530 PMC4070281

[5] S. Cauchemez , C. A. Donnelly , C. Reed , et al., “Household Transmission of 2009 Pandemic Influenza A (H1N1) Virus in the United States,” The New England Journal of Medicine 361 (2009): 2619–2627. 20042753 10.1056/NEJMoa0905498 PMC3840270

[6] C. Viboud , P. Y. Boëlle , S. Cauchemez , et al., “Risk Factors of Influenza Transmission in Households,” The British Journal of General Practice 54 (2004): 684–689. 15353055 PMC1326070

[7] H. Nishiura and H. Oshitani , “Household Transmission of Influenza (H1N1‐2009) in Japan: Age‐Specificity and Reduction of Household Transmission Risk by Zanamivir Treatment,” The Journal of International Medical Research 39 (2011): 619–628. 21672367 10.1177/147323001103900231

[8] A. M. Fry , D. Goswami , K. Nahar , et al., “Effects of Oseltamivir Treatment of Index Patients With Influenza on Secondary Household Illness in an Urban Setting in Bangladesh: Secondary Analysis of a Randomised, Placebo‐Controlled Trial,” The Lancet Infectious Diseases 15 (2015): 654–662. 25788164 10.1016/S1473-3099(15)70041-1

[9] S. Ng , B. J. Cowling , V. J. Fang , et al., “Effects of Oseltamivir Treatment on Duration of Clinical Illness and Viral Shedding and Household Transmission of Influenza Virus,” Clinical Infectious Diseases 50 (2010): 707–714. 20121573 10.1086/650458 PMC2840043

[10] E. Goldstein , B. J. Cowling , J. J. O'Hagan , et al., “Oseltamivir for Treatment and Prevention of Pandemic Influenza A/H1N1 Virus Infection in Households, Milwaukee, 2009,” BMC Infectious Diseases 10 (2010): 1–7. 20642862 10.1186/1471-2334-10-211 PMC2919545

[11] N. Hirotsu , S. Yutaka , and H. Takahiro , “The Effect of Neuraminidase Inhibitors on Household Transmission in Japanese Patients With Influenza A and B Infection: A Prospective, Observational Study,” Influenza and Other Respiratory Viruses 13 (2019): 123–132. 29989680 10.1111/irv.12590 PMC6379638

[12] F. G. Hayden , N. Sugaya , N. Hirotsu , et al., “Baloxavir Marboxil for Uncomplicated Influenza in Adults and Adolescents,” The New England Journal of Medicine 379 (2018): 913–923. 30184455 10.1056/NEJMoa1716197

[13] Y.‐A. Heo , “Baloxavir: First Global Approval,” Drugs 78 (2018): 693–697. 29623652 10.1007/s40265-018-0899-1

[14] M. G. Ison , S. Portsmouth , Y. Yoshida , et al., “Early Treatment With Baloxavir Marboxil in High‐Risk Adolescent and Adult Outpatients With Uncomplicated Influenza (CAPSTONE‐2): A Randomised, Placebo‐Controlled, Phase 3 Trial,” The Lancet Infectious Diseases 20 (2020): 1204–1214. 32526195 10.1016/S1473-3099(20)30004-9

[15] J. Baker , S. L. Block , B. Matharu , et al., “Baloxavir Marboxil Single‐Dose Treatment in Influenza‐Infected Children: A Randomized, Double‐Blind, Active Controlled Phase 3 Safety and Efficacy Trial (miniSTONE‐2),” The Pediatric Infectious Disease Journal 39 (2020): 700–705. 32516282 10.1097/INF.0000000000002747 PMC7360097

[16] T. Umemura , Y. Mutoh , T. Kawamura , et al., “Efficacy of Baloxavir Marboxil on Household Transmission of Influenza Infection,” Journal of Pharmaceutical Health Care and Sciences 6 (2020): 1–6. 33014405 10.1186/s40780-020-00178-4 PMC7528271

[17] T. Komeda , T. Takazono , N. Hosogaya , et al., “Comparison of Household Transmission of Influenza Virus From Index Patients Treated With Baloxavir Marboxil or Neuraminidase Inhibitors: A Health Insurance Claims Database Study,” Clinical Infectious Diseases 72 (2021): 859–867. 10.1093/cid/ciaa1622 33103200

[18] S. Miyazawa , T. Takazono , N. Hosogaya , et al., “Comparison of Intra‐Familial Transmission of Influenza Virus From Index Patients Treated With Baloxavir Marboxil or Oseltamivir Using an Influenza Transmission Model and a Health Insurance Claims Database,” Clinical Infectious Diseases 75 (2022): 927–935. 35100617 10.1093/cid/ciac068 PMC9522426

[19] H. Ikematsu , F. G. Hayden , K. Kawaguchi , et al., “Baloxavir Marboxil for Prophylaxis Against Influenza in Household Contacts,” The New England Journal of Medicine 383 (2020): 309–320. 32640124 10.1056/NEJMoa1915341

[20] M. M. Saito , N. Hirotsu , H. Hamada , et al., “Reconstructing the Household Transmission of Influenza in the Suburbs of Tokyo Based on Clinical Cases,” Theoretical Biology & Medical Modelling 18 (2021): 1–10. 33568160 10.1186/s12976-021-00138-x PMC7873673

[21] G. Zou , “A Modified Poisson Regression Approach to Prospective Studies With Binary Data,” American Journal of Epidemiology 159 (2004): 702–706. 15033648 10.1093/aje/kwh090

[22] N. Yoshii , Y. Tochino , M. Fujioka , et al., “The Comparison of the Efficacy of Baloxavir and Neuraminidase Inhibitors for Patients With Influenza a in Clinical Practice,” Internal Medicine 59 (2020): 1509–1513. 32536677 10.2169/internalmedicine.4117-19 PMC7364256

[23] V. Taieb , H. Ikeoka , F.‐F. Ma , et al., “A Network Meta‐Analysis of the Efficacy and Safety of Baloxavir Marboxil Versus Neuraminidase Inhibitors for the Treatment of Influenza in Otherwise Healthy Patients,” Current Medical Research and Opinion 35 (2019): 1355–1364. 30810054 10.1080/03007995.2019.1584505

[24] V. Taieb , H. Ikeoka , P. Wojciechowski , et al., “Efficacy and Safety of Baloxavir Marboxil Versus Neuraminidase Inhibitors in the Treatment of Influenza Virus Infection in High‐Risk and Uncomplicated Patients—A Bayesian Network Meta‐Analysis,” Current Medical Research and Opinion 37 (2021): 225–244. 33079575 10.1080/03007995.2020.1839400

[25] World Health Organization Writing Group , “Nonpharmaceutical Interventions for Pandemic Influenza, International Measures,” Emerging Infectious Diseases 12, no. 1 (2006): 81–87. 16494722 10.3201/eid1201.051370 PMC3291414

[26] N. Hirotsu , W. Koji , and O. Hitoshi , “Risk Factors of Household Transmission of Pandemic (H1N1) 2009 Among Patients Treated With Antivirals: A Prospective Study at a Primary Clinic in Japan,” PLoS ONE 2 (2012): e31519. 10.1371/journal.pone.0031519 PMC3281072 22359599

[27] Y. Chong , N. Kawai , N. Tani , et al., “Virological and Clinical Outcomes in Outpatients Treated With Baloxavir or Oseltamivir: A Japanese Multicenter Study in the 2019–2020 Influenza Season,” Antiviral Research 192 (2021): 105092. 34052230 10.1016/j.antiviral.2021.105092

[28] E. Takashita , C. Kawakami , R. Ogawa , et al., “Influenza a (H3N2) Virus Exhibiting Reduced Susceptibility to Baloxavir due to a Polymerase Acidic Subunit I38T Substitution Detected From a Hospitalised Child Without Prior Baloxavir Treatment, Japan, January 2019,” Euro Surveillance 12 (2019): 1900170. 10.2807/1560-7917.ES.2019.24.12.1900170 PMC6440584 30914078

[29] E. Takashita , M. Ichikawa , H. Morita , et al., “Human‐to‐Human Transmission of Influenza A (H3N2) Virus With Reduced Susceptibility to Baloxavir, Japan, February 2019,” Emerging Infectious Diseases 25, no. 11 (2019): 2108–2111. 31436527 10.3201/eid2511.190757 PMC6810216

[30] J. Harding , C. Bernasconi , S. Williams , et al., “Investigating the Transmission of Baloxavir‐Resistant Influenza Viruses From Treated Index Patients to Untreated Household Contacts in the BLOCKSTONE Study,” Influenza and Other Respiratory Viruses 17, no. 1 (2023): e13079. 36702798 10.1111/irv.13079 PMC9849088

[31] J. Asher , A. Lemenuel‐Diot , M. Clay , et al., “Novel Modelling Approaches to Predict the Role of Antivirals in Reducing Influenza Transmission,” PLoS Computational Biology 19 (2023): e1010797. 36608108 10.1371/journal.pcbi.1010797 PMC9876374

[32] Z. du , C. Nugent , A. P. Galvani , R. M. Krug , and L. A. Meyers , “Modeling Mitigation of Influenza Epidemics by Baloxavir,” Nature Communications 11 (2020): 2750. 10.1038/s41467-020-16585-y PMC7265527 32487990

