# Supplementary material for: Comparative Effectiveness of Baloxavir Marboxil and Oseltamivir Treatment in Reducing Household Transmission of Influenza: A Post Hoc Analysis of the BLOCKSTONE Trial
Source: Influenza Other Respir Viruses. 2024 May 6;18(5):e13302. doi: 10.1111/irv.13302 (PMC11070769; doi:10.1111/irv.13302)
Supplement: Supplementary file 1 — Figure S1. Avoidance ratio of intra‐household ill cases based on modified Poisson regression model. Figure S2. Serial interval and assumed infectious period in simulation‐applicable population. Table S1. Demographic characteristics of the index cases (simulation‐applicable population). Table S2. Demographic characteristics of the household contacts (simulation‐applicable population). Table S3. Demographic characteristics of the ill household contacts who participating in the clinical trial or questionnaire survey. Table S4. Estimated parameters in days for the transmission profiles of index cases in the simulation‐applicable population. Table S5. Secondary attack rate based on Poisson regression model for household contacts participating in the clinical trial or questionnaire survey. [file IRV-18-e13302-s002.docx]

**Supplementary Methods**

**Transmission profile simulation**

Using previously reported methods [1], we estimated the parameters determining the transmission profile. The serial interval, which is the time between the illness onset of the index case and illness onset of the secondary case, was assumed to be the sum of the time until secondary infection occurred (waiting time) and the incubation period. It was assumed that the waiting time followed a uniform distribution with the infective period as an upper limit value (truncated uniform distribution), and the incubation period followed a gamma distribution [2]. Mathematically, this assumption of waiting time corresponds to the assumption that infectivity was constant during the infective period, and the distribution of waiting time and infective period were also estimated. Consequently, the serial interval was expressed as a convolution of the distributions. Because the serial interval was observed in this study, we estimated the distributions of waiting time and incubation period using the maximum likelihood estimation method fitting to the serial interval. We also estimated the distribution of the symptomatic period assuming a gamma distribution, and the extended infective period was estimated by subtracting the symptomatic period from the infectious period.

**References**

1. Saito, M. M, Hirotsu, N, Hamada, H, et al. Reconstructing the household transmission of influenza in the suburbs of Tokyo based on clinical cases. Theor Biol Med Model **2021**; 18:1–10.
2. Johnson NL, Samuel K, Narayanaswamy B. Continuous univariate distributions, volume 2. Vol. 289. John Wiley & Sons, **1995**.

**Supplementary Figure 1. Avoidance ratio of intra-household ill cases based on modified Poisson regression model**

CI, Confidence interval; BXM, baloxavir marboxil; OTV, oseltamivir

BXM was used as a reference against OTV to calculate the avoidance ratio.

† Modified Poisson regression model with the presence or absence of intra-household ill cases as the outcome and the treatment drugs of the index patientindex case as the fixed effect.

‡ Modified Poisson regression model with the presence or absence of intra-household ill cases as the outcome, treatment of the index patientindex case as the fixed effect, and age, influenza vaccination status within the previous six months of the index case and household size as covariates.

In subgroup analysis by age and influenza subtype, age was excluded from the analysis model.

**Supplementary Figure 2.** Serial interval and assumed infectious period in simulation-applicable population


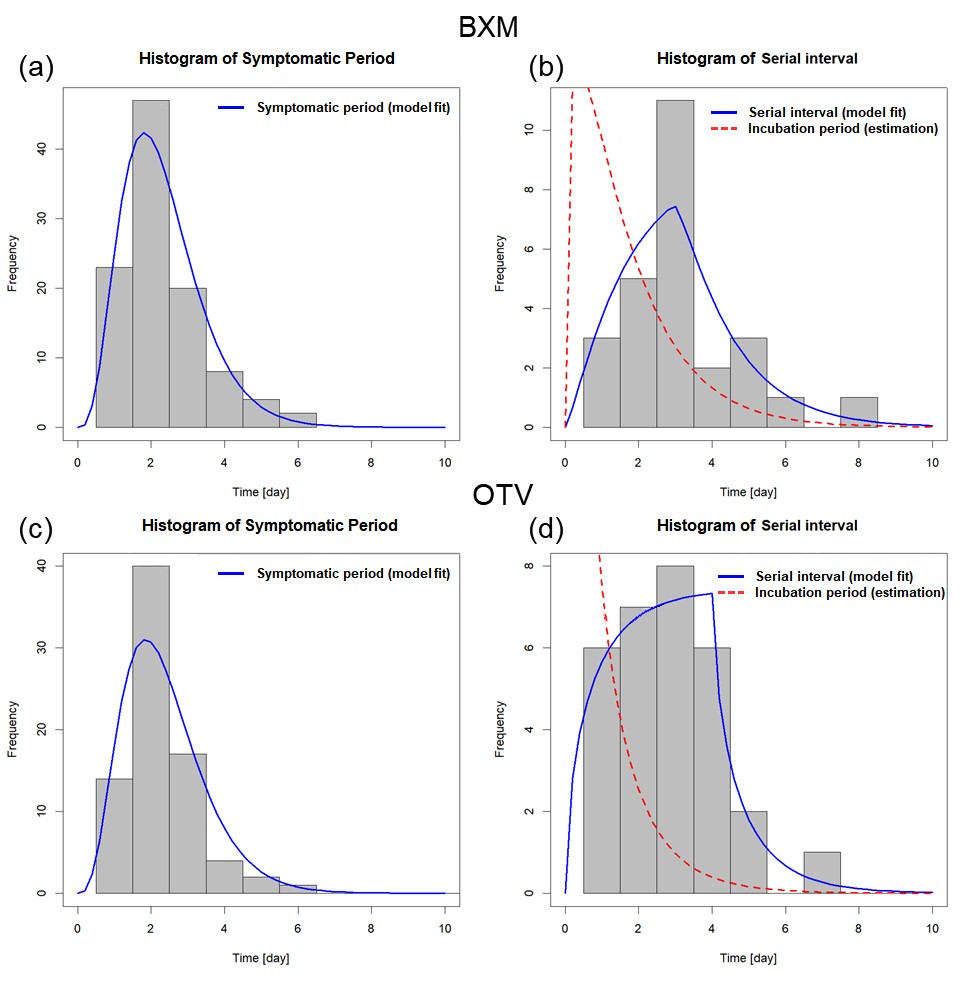


Symptomatic period and serial intervals for the dataset of BXM- or OTV-treated index case. (a) Histogram of the symptomatic period and its fit to gamma distribution for baloxavir recipients. (b) Histogram of the observed serial interval compared to the estimated interval (blue curve), where the estimated serial interval is the sum of the waiting time and gamma-distributed incubation period (red curve) for baloxavir recipients. (c) and (d) are the corresponding data for oseltamivir recipients.

**Supplementary Table 1.** Demographic characteristics of the index cases (simulation-applicable population)

|  |  | BXM (N=84)  n (%) | OTV (N=58)  n (%) | Overall (N=142)  n (%) |
| --- | --- | --- | --- | --- |
| Sex | Male | 44 (52.4) | 31 (53.4) | 75 (52.8) |
| Age | < 12 years | 45 (53.6) | 55 (94.8) | 100 (70.4) |
|  | ≥ 12 to < 64 years | 35 (41.7) | 2 (3.4) | 37 (26.1) |
|  | ≥ 64 years | 4 (4.8) | 1 (1.7) | 5 (3.5) |
| Vaccination* | Yes | 25 (29.8) | 24 (41.4) | 49 (34.5) |
| Influenza virus type | Type A | 84 (100.0) | 58 (100.0) | 142 (100.0) |
|  | - A/H1N1pdm09 | 36 (42.9) | 30 (51.7) | 66 (46.5) |
|  | - A/H3NX | 48 (57.1) | 28 (48.3) | 76 (53.5) |
| Occupation | Worker | 11 (13.1) | 1 (1.7) | 12 (8.5) |
|  | Student | 69 (82.1) | 50 (86.2) | 119 (83.8) |
|  | Neither | 4 (4.8) | 7 (12.1) | 11 (7.7) |
| Household size except for index case excluded | < 3 | 23 (27.4) | 16 (27.6) | 39 (27.5) |
|  | ≥ 3 | 61 (72.6) | 42 (72.4) | 103 (72.5) |

***** Influenza vaccination status within the previous six months. BXM, baloxavir marboxil; OTV, oseltamivir

**Supplementary Table 2.** Demographic characteristics of the household contacts (simulation-applicable population)

|  |  | Clinical trial | | Questionnaire | | Overall | |
| --- | --- | --- | --- | --- | --- | --- | --- |
|  |  | BXM  N = 92  n (%) | OTV  N = 65  n (%) | BXM  N = 120  n (%) | OTV  N = 73  n (%) | BXM  N = 212  n (%) | OTV  N = 138  n (%) |
| Sex | Male | 17 (18.5) | 11 (16.9) | --- | --- | 17 (8.0) | 11 (8.0) |
|  | Female | 75 (81.5) | 54 (83.1) | --- | --- | 75 (35.4) | 54 (39.1) |
|  | Missing | --- | --- | 120 (100.0) | 73 (100.0) | 120 (56.6) | 73 (52.9) |
| Age | < 12 years | 5 (5.4) | 6 (9.2) | 9 (7.5) | 17 (23.3) | 14 (6.6) | 23 (16.7) |
|  | ≥ 12 to ≤ 64 years | 81 (88.0) | 55 (84.6) | 9 (7.5) | 3 (4.1) | 90 (42.5) | 58 (42.0) |
|  | ≥ 65 years | 6 (6.5) | 4 (6.2) | 0 | 0 | 6 (2.8) | 4 (2.9) |
|  | Missing | --- | --- | 102 (85.0) | 53 (72.6) | 102 (48.1) | 53 (38.4) |
| Time from onset of influenza virus infection of index case to informed consent of household contact | < 24 hours | 65 (70.7) | 51 (78.5) | --- | --- | 65 (30.7) | 51 (37.0) |
|  | ≥ 24 to ≤ 48 hours | 27 (29.3) | 14 (21.5) | --- | --- | 27 (12.7) | 14 (10.1) |
|  | Missing | --- | --- | 120 (100.0) | 73 (100.0) | 120 (56.6) | 73 (52.9) |
| Vaccination* | Yes | 28 (30.4) | 24 (36.9) | --- | --- | 28 (13.2) | 24 (17.4) |
|  | No | 64 (69.6) | 41 (63.1) | --- | --- | 64 (30.2) | 41 (29.7) |
|  | Missing | --- | --- | 120 (100.0) | 73 (100.0) | 120 (56.6) | 73 (52.9) |
| Influenza virus type of index case | Type A | 92 (100.0) | 65 (100.0) | 120 (100.0) | 73 (100.0) | 212 (100.0) | 138 (100.0) |
|  | - A/H1 | 41 (44.6) | 34 (52.3) | 42 (35.0) | 42 (57.5) | 83 (39.2) | 76 (55.1) |
|  | - A/H3 | 51 (55.4) | 31 (47.7) | 78 (65.0) | 31 (42.5) | 129 (60.8) | 62 (44.9) |
| Occupation | Worker | 60 (65.2) | 44 (67.7) | 5 (4.2) | 2 (2.7) | 65 (30.7) | 46 (33.3) |
|  | Student | 5 (5.4) | 7 (10.8) | 11 (9.2) | 14 (19.2) | 16 (7.5) | 21 (15.2) |
|  | Neither | 27 (29.3) | 14 (21.5) | 2 (1.7) | 5 (6.8) | 29 (13.7) | 19 (13.8) |
|  | Missing | --- | --- | 102 (85.0) | 52 (71.2) | 102 (48.1) | 52 (37.7) |

* Influenza vaccination status within the previous six months

Age and occupation of household contacts who participated in the questionnaire survey were available if they were ill. BXM, baloxavir marboxil; OTV, oseltamivir

**Supplementary Table 3.** Demographic characteristics of the ill household contacts who participating in the clinical trial or questionnaire survey

|  |  | BXM | | OTV | | Overall | |
| --- | --- | --- | --- | --- | --- | --- | --- |
|  |  | Clinical trial  N = 18  n (%) | Questionnaire  N = 17  n (%) | Clinical trial  N = 18  n (%) | Questionnaire  N = 19  n (%) | Clinical trial  N = 36  n (%) | Questionnaire  N = 36  n (%) |
| Age | < 12 years | 0 | 9 (52.9) | 1 (5.6) | 16 (84.2) | 1 (2.8) | 25 (69.4) |
|  | ≥ 12 to ≤ 64 years | 18 (100) | 8 (47.1) | 17 (94.4) | 2 (10.5) | 35 (97.2) | 10 (27.8) |
|  | ≥ 65 years | 0 | 0 | 0 | 0 | 0 | 0 |
|  | Missing | 0 | 0 | 0 | 1 (5.3) | 0 | 0 |
| Influenza virus type of index case | Type A | 18 (100) | 17 (100) | 18 (100) | 19 (100) | 36 (100) | 36 (100) |
|  | - A/H1N1pdm09 | 6 (33.3) | 6 (35.3) | 5 (27.8) | 13 (68.4) | 11 (30.6) | 19 (52.8) |
|  | - A/H3NX | 12 (66.7) | 11 (64.7) | 13 (72.2) | 6 (31.6) | 25 (69.4) | 17 (47.2) |
|  | Type B | 0 | 0 | 0 | 0 | 0 | 0 |
|  | Mixed infection | 0 | 0 | 0 | 0 | 0 | 0 |
| Occupation | Worker | 11 (61.1) | 6 (35.3) | 14 (77.8) | 1 (5.3) | 25 (69.4) | 7 (19.4) |
|  | Student | 1 (5.6) | 9 (52.9) | 1 (5.6) | 14 (73.7) | 2 (5.6) | 23 (63.9) |
|  | Neither | 6 (33.3) | 2 (11.8) | 3 (16.7) | 4 (21.1) | 9 (25.0) | 6 (16.7) |

BXM, baloxavir marboxil; OTV, oseltamivir

Clinical trial: Household contacts participated in the BLOCKSTONE trial were enrolled.

Questionnaire: Household contacts participated in this study via questionnaire survey were enrolled.**Supplementary Table 4.** Estimated parameters in days for the transmission profiles of index cases in the simulation-applicable population.

|  |  | BXM | OTV |
| --- | --- | --- | --- |
| Symptomatic Period | |  |  |
|  | 95% CI | 1.00-5.00 | 1.00-5.05 |
|  | Median | 2.00 | 2.00 |
|  | Mean | 2.32 | 2.38 |
|  | Gamma dist. Shape | 4.59 | 4.48 |
|  | Gamma dist. Scale | 0.50 | 0.53 |
| Serial Interval | |  |  |
|  | 95% CI | 1.00-6.75 | 1.00-5.55 |
|  | Median | 3.00 | 3.00 |
| Assumed infective period | | |  |
|  | point estimate | 2.97 | 4.03 |
| Incubation period | |  |  |
|  | 95% CI | 0.08-5.36 | 0.00-3.42 |
|  | Mean | 1.62 | 0.72 |
|  | Gamma dist. Shape | 1.28 | 0.56 |
|  | Gamma dist. Scale | 1.26 | 1.28 |
| Extended infective period | |  |  |
|  | point estimate | 0.65 | 1.65 |

Units of all parameters are days.

CI, confidence interval; BXM, baloxavir marboxil; OTV, oseltamivir

**Supplementary Table 5.** Secondary attack rate based on Poisson regression model for household contacts participating in the clinical trial or questionnaire survey.

| Population | Treatment | Number of household contacts | Number of ill cases | Unadjusted SAR |
| --- | --- | --- | --- | --- |
|  |  |  |  |  |
| Male, < 12 years | BXM | 68 | 13 | 15.9% |
|  | OTV | 77 | 18 | 18.0% |
| Male, ≥ 12 years | BXM | 74 | 11 | 10.8% |
|  | OTV | 1 | 0 | 0.0% |
| Female, < 12 years | BXM | 74 | 6 | 5.8% |
|  | OTV | 71 | 19 | 20.4% |
| Female, ≥ 12 years | BXM | 41 | 5 | 10.2% |
|  | OTV | 4 | 0 | 0.0% |

SAR, secondary attack rate; CI, confidence interval; BXM, baloxavir marboxil; OTV, oseltamivir

BXM was used as a reference against OTV to calculate the SAR rate.

Poisson regression model with the number of newly ill persons among the population in the household as the outcome, treatment of the index case as the fixed effect, and household size among the population, except for the index case, as offsets.
