# Supplementary material for: Comparative Effectiveness of Baloxavir Marboxil and Oseltamivir Treatment in Reducing Household Transmission of Influenza: A Post Hoc Analysis of the BLOCKSTONE Trial
Source: Influenza Other Respir Viruses. 2024 May 6;18(5):e13302. doi: 10.1111/irv.13302 (PMC11070769; doi:10.1111/irv.13302)
Supplement: Supplementary file 2 — Data S1 Supporting Information. [file IRV-18-e13302-s001.docx]

**Dear family members or cohabitants of the participants**

**Request for your cooperation in the research study on influenza**

We are requesting that household contacts of index case who participated in the "Phase 3, Randomized, Double-Blind, Placebo-Controlled Study to Evaluate the Inhibitory Effect of a Single Dose of Baloxavir Marboxil on the Development of Influenza Virus Infection", to answer the following survey regarding influenza.

Title: Study of Influenza Transmission - Questionnaire-Based Survey

The purpose of this survey is to examine the routes and influencing factors of influenza transmission within families, and to obtain information on the prevalence of influenza through extra-familial transmission. If you agree to participate in this study, we will ask you to provide us with four items of information on the household contacts. Please refer to the attached questionnaire for details of the questions to be asked. The study participants will record the results of their questions on the attached questionnaire, which they will bring to the hospital on the last day of their visit. The total time required for the questionnaire is approximately 5 minutes. We will not ask you to pay any fees for answering the survey questions. We will not ask you to pay for your participation in the study, nor will we give you a fee for your participation.

You are free to decide whether or not to participate, and you will not be disadvantaged in any way if you do not participate. The collected questionnaire responses will be anonymized before being sent from the data collector to the research administrator. Therefore, the research administrator will analyze the data without having any information that can identify the respondents.

Shionogi is deeply aware that personal information should be handled carefully based on the principle of respect for human rights, and will comply with applicable laws and regulations regarding personal information, and will handle personal information legally and appropriately based on the Privacy Policy.

If you agree to participate in this study, please sign the attached consent form. After that, please answer the questions from the subjects. We appreciate your understanding and cooperation in this research study.

**A Study of Influenza Infections in Household**

**--Survey research based on questionnaire--**

**Consent Form**

【Person in question】

I have received an explanation of the items described in the explanatory document regarding the "Study of Influenza Transmission - Questionnaire-Based Survey" and I have agreed to participate in this study.

I have received a copy of the Explanatory Document and this Consent Form.

Participant 1 Year Month Day Signature：

Participant 2 Year Month Day Signature：

Participant 3 Year Month Day Signature：

Participant 4 Year Month Day Signature：

Participant 5 Year Month Day Signature：

Participant 6 Year Month Day Signature：

Participant 7 Year Month Day Signature：

Participant 8 Year Month Day Signature：

Participant 9 Year Month Day Signature：

Participant 10 Year Month Day Signature：

【Guardian】

I have received and accepted the information contained in the Explanatory Document on behalf of the Participant for the reasons stated below, and I consent to the Participant's participation in this study.

I have received a copy of the Explanatory Document and this Consent Form.

Reasons for difficulty in obtaining consent from the individual：

Participant 11 Year Month Day Signature：

（Relationship to the participant： ）

Reasons for difficulty in obtaining consent from the individual：

Participant 12 Year Month Day Signature：

（Relationship to the participant： ）

Reasons for difficulty in obtaining consent from the individual：

Participant 13 Year Month Day Signature：

（Relationship to the participant： ）

Reasons for difficulty in obtaining consent from the individual：

Participant 14 Year Month Day Signature：

（Relationship to the participant： ）

Reasons for difficulty in obtaining consent from the individual：

Participant 15 Year Month Day Signature：

（Relationship to the participant： ）

**--** **Study of Influenza Transmission - Questionnaire-Based Survey --**

**Questionnaire**

- **Please answer the following questions about your individual situation regarding influenza infection. Please check "No" in Q1 if there are no symptoms in Q1 by the last visit to the hospital for those who have taken the study drug. If you have any of the symptoms in Q1, please check "Yes" in Q1 and answer the subsequent items based on the information up to the date of the last visit to the hospital for the person who took the investigational drug. 　Date of response:**

| Q1. Did you develop influenza-like symptoms such as a body temperature (axillary temperature*1) of 37.5°C or higher and either "cough" or "runny/stuffy nose"? | □ Yes  □ No |
| --- | --- |
| If you checked "No" in Q1, the question is closed.  If you checked "Yes" in Q1, please answer Q2-1 below. | |
| Q2-1. Please indicate your age group when you developed influenza-like symptoms such as body temperature (axillary temperature) of 37.5°C or higher and either "cough" or "runny/stuffy nose". | □ Under 12 years old  □ 12 years old or older but under 65 years old  □ 65 years old or older |
| Q2-2. Please indicate your occupation when you developed the influenza-like symptoms listed in Q2-1. | □ Student*2, 4  □ Worker*3, 4  □ Other than above |
| Q2-3. When did you develop the influenza-like symptoms listed in Q2-1 (morning or afternoon)? | Date:  □ AM  □ PM  □ Unknown |
| Q3. By the date of the subject's last visit to the hospital, did you confirm that the body temperature (axillary temperature) had reached a normal body temperature (<37.0°C)? | □ Confirmed  □ Not confirmed  □ Not back to normal body temperature |
| If you checked "not confirmed" or "not returned to normal body temperature" in Q3, please answer Q4. | |
| Q4. When was the date (morning or afternoon) that you confirmed that your body temperature (axillary temperature) returned to normal (<37.0°C)? | Date:  □ AM  □ PM  □ Unknown |

*1 Body temperature under the armpit.

*2 Students include those enrolled in nursery schools, kindergartens, elementary schools, junior high schools, high schools, vocational schools, universities, and graduate schools.

*3 Workers include company employees, self-employed workers, part-time workers, and others who have contact with others other than family members and cohabitants while commuting to and from work.

*4 If you are both a student and a worker, please select "student".

*5 The person who signed the consent form should answer the question, and if the answer is incorrect, that person should correct it.
